# What Fools These Mortals Be

**Published:** 1885-04

**Authors:** Ben. H. Pratt


### THE BISTOURY.

Vol. XXI. ELMIRA, N. Y., APRIL, 1885. No. 1.

### Contributions.

### "WHAT FOOLS THESE MORTALS BE."

BEN. H. PRATT.

Nine people out of ten are ashamed of their ages. A more ridiculous truth doesn't mar our social economy. All women and nearly all men are sensitive as to their years. Ask a lady her height, her weight, the length of her hair, the cost of her wardrobe, or any direct personal question, and she may deem the inquiry rather brusque or somewhat impertinent or even impudent, but she will recover her equanimity soon and feel rather complimented by your curiosity. At the worst, you are but temporarily her enemy, other things being favorable toward mutual friendship. But ask her how old she is and you will never be forgiven. She will remember it to her last conscious moment. She will think of it every time she sees you; every time your name is spoken in her presence. You can never overcome such an imprudence. She may endure your presence, may become reconciled to your society, may tolerate your visits, may suffer your courtship, may even accompany you to the alter and vow to love, honor, and obey you, but she does not, cannot, forget the indiscretion, nor fully forgive you. It is the cause of more domestic unhappiness than anything except rum. It alienates more men and women than do homely faces, slovenly habits, slouchy toilets, snaggy teeth, or bad breaths. It is the social sin that cannot be pardoned.

Why is it ?

What under the sun is there so dreadful about a desire to know one's age ?

What matters it, if one be twenty, or five and twenty, or five and thirty, or fifty or seventy years old ?

Is there any disgrace attaching to one's years ?

Does one's age stand in the way of securing any special boon in life ?

Isn't it ridiculous how people will lie about their ages—otherwise good, church-going, christian models of society, and eminent in the church ?"

These queries have puzzled me ever since I can remember. I have looked and studied in vain for a solution of these, the silliest of all the social phenomena that are presented. Don't women realize how very weak minded this habit of trying to conceal their ages makes them appear ? The mere suggestion as to what may possibly be the age of one not present, sets all those within hearing into a perfect agony of dread lest something may be said as to their own age, or lest somebody may intimate that their age is known and may either be blurted out, or revealed by a course of comparative reasoning, or disclosed by some slip of the tongue or in some way become known. The subject is sure to be dropped, if that be possible, or the line of thought and conversation is assiduously and cleverly directed into some other channel. Parties have been broken up in the midst of the jolliest festivities by the possibility of somebody's being on the point of declaring somebody's age.

When, where and how did it become such a heinous crime to seek to know or to reveal a lady's age ? History does not furnish the information, for the silly dread seems to antedate all history. The dread doesn't have to be learned, for children possess it without being taught. Our small misses, who have not, as yet, reached the dignity of sitting in grown people's chairs at juvenile tea parties, will giggle and simper over, and guess at each other's ages with all the smirks and frowns and elevation of eyebrows and indignant grimace, and wriggling of shoulders, and elevation of noses and depression of the corners of their mouths that characterize their elders around the otherwise festive board. It probably began in the Garden of Eden. We are not told how old Eve was when she first appeared before Adam, and possibly Adam couldn't tell how long he slept after he was beribbed, for his sleep we are told was a "deep" one. This, possibly, was what gave Eve her first opportunity to tease Adam. He naturally desired to know how long he had slept, and not knowing any better way of getting at it than by learning the age of his new companion, man-like he was so unfortunate as to ask how old she was. The feminine perversity here had its earliest opportunity, and she evaded the question as long as possible, and perceiving the effect it had upon Adam—for it probably made him mad to be thwarted in an affair of so little moment to her and of considerable auto-biographical interest to him—discovered that she had one advantage over her lord, and meant to hang on to it. This disposition doubtless grew with her growth and strengthened with her strength; became one of the ruling passions of her life. She knew that Adam could never know how old she was unless she told him. There were no "family records" in those days, nor any sewing societies, nor ladies' aid meetings, nor tea parties, nor any kind neighbors to impart the secret. It was her very own, a charge to keep, and she kept it. It doubtless caused the little domestic differences in the family that brought about little tiffs, and now and then a temporary separation, when Eve, instead of watching Adam dig potatoes or fight the weevil in the wheat, betook herself to some one of her favorite nooks in the garden and communed with herself. These communings were of that brooding sort that ever invokes the devil, and that personage doubtless knew his opportunity when he persuaded her to bite into the forbidden fruit. She doubtless did it to spite Adam for wanting to know how old she was, and when he got through his chores, and felt like making up with his pettish wife, she, in her sex-like way, pouted, and chucked the apple at him, when he, seeing the marks of her perfect little teeth in the delicious fruit, seized and put the sweet souvenir to his lips to kiss the spot she had made sacred to him, and finding it "pleasant to the taste," ate and fell. There is little doubt that Milton, when he sang "of man's first disobedience and the fruit of that forbidden tree whose mortal taste brought death into the world, and all our woe," did not go back quite far enough for the real origin of the trouble. Milton, however, had few opportunities to know how thoroughly incorporated within the female mind was this innate desire to conceal her age. He ascribes the fall to her curiosity, or to her desire to be amiable in her association with the talking serpent, or to her incredulity as to what Adam had said about the fruit of that one particular tree, or to her belief that he was guying her, or that he wanted that special fruit for a state occasion, and didn't care to have her picking at it until he pronounced it fully ripe. Milton meant well, but he erred in his judgment of matters with which he chanced to be unfamiliar. Then, he was blind, and lost many opportunities for observation that a seeing man could not have escaped. There isn't much doubt now, among Sociologists, that the origin of this dread of declaring one's age not only had its origin in Eden, but that Adam's fall, whereby we "sinned all," may assuredly be traced back to Eve's persistence in not disclosing her years.

This was all the more inexcusable in Eve because she was young, with a fair chance of never growing any the worse for age. She had never seen any old women. She didn't realize that she would ever be old and wrinkled, and probably never would have been but for her indiscretion. She was doubtless destined to live forever, and it isn't at all reasonable that she was to become any less comely because of age. She had no precedent to go by, so that it was pure perverseness that made her act in that very ridiculous manner. Her children inherited this trait, this silly persistence in concealing their age from everybydy, and this accounts, in a large measure for the unfortunate phenomenon over which men have philosophized ever since the world was inhabited by men.

It is difficult to account for the phenomena upon any other hypothesis. Neither women nor men can gain anything by concealing and lying about their ages. There are certain plain and unmistakable signs of age. It doesn't require an expert to tell, within a very few years, at most, and generally within a few months, what the age of a man or woman is, and even where the record of birth is wanting, ages are at least approximated, if they are not absolutely known.

A silly idea prevails, with some young people, that a knowledge of their ages may interfere with their matrimonial prospects, and I more than suspect that this notion does more than anything else to maintain the desire to conceal one's age. It is a mistake, however, for the pretence is sure to be sooner or later discovered, and the deceit thus practiced only reacts to the disadvantage of the one practicing it. Men don't marry women nor women men for their age, nor for their youth, but because they desire each other's companionship. Age has nothing to do with it provided other circumstances are favorable. Men don't take women as boys used to swop jack-knives, "unsight and unseen," but after intimate and more or less protracted association. When two people conclude to be married they are not to be separated by any suddenly gained knowledge that the individual to whom the pledge has been made is a few years older or younger than had been supposed. Courtships are not deliberately entered upon because ages are precisely suitable, each to each, nor are two persons deterred from making matrimonial contracts because of a newly discovered difference in the age of either from what had been previously supposed.

Nor is there any social disgrace attaching to years. All ages have their distinct and peculiar joys. The young may think that the old have no fun, and may dread the thought of growing old, or that people should know that they are growing old. This is so shallow a view of life that only the weak minded entertain it. The facts are, and they prove themselves constantly to the observing, that the older men and women grow, the more real enjoyment they have. They do not enjoy the same pleasures that fascinate the young, but their joys are far more substantial. They are very foolish young persons who imagine they are the only happy ones. They are short sighted, indeed, if they do not recognize wherein the enjoyments of those advanced in life are more substantial, more enduring and far more free from annoyances and perplexities than are the lives of young people. It must be remembered that while the older ones know precisely what their joys were when young, they also know what they are in maturer life, while the younger ones have no knowledge whatever of the joys that enter into the lives of their elders. But even admitting that the young are satisfied that age brings nothing but misery, that alone could not account for the silly desire of any to appear younger than they are.

Perhaps young people do not realize how very simple and mawkish they appear when trying to convey the idea that they are younger than they actually are. Then among the funniest of all situations that can be conceived, is the performance of a spinster, say forty years old, whom some flatterer, in a very delicate and quite indirect way, declares to be no older than twenty-five. Do these poor souls actually believe that any sane person could make so gross an error of judgment? No, they do not, but they presume to believe—and in many cases women do actually believe—that they are the only exceptions to the general rule in all the world; that their personal appearances do not correspond with their ages; that while all other men and women show to all the world just how old they are, each particular youngish woman cherishes the very comfortable idea that nobody could ever tell her age from her looks.

There never was a more fallacious notion than this entertained by a human being. The marks of age in the human kind are more absolutely and unmistakably impressed upon the physical being than in the case of any other creature, and he or she who doesn't recognize this fact, deceives himself or herself cruelly. There is but one explanation of the phenomenon of self-deception, and this is the natural vanity of the race. It has been said that "all men think all men mortal but themselves." It might be added that all men and women see defects in everybody except themselves; that all are continually prone to excuse their own conduct and criticise that of all others; that all women imagine that all other women are growing old, while they plume themselves upon the belief that they remain in *statu quo* as to the signs of advancing age. There are few exceptions to this remarkable impression; so few, that the lady who openly declares her real age and admits that she is getting older in looks as well as in years, is pointed out as a very remarkable person.

It is one of the most beautiful phenomena of social life to watch a lady cheerfully and gracefully and consciously growing old.. She sheds about her a halo of happiness and contentment that makes her presence a benison. Her husband adores her, her children revere her, her friends love her and her acquaintances pay court to her constantly. She is known far and wide throughout her neighborhood, and honored by hundreds whom she never knew. Such a woman never grows old in anything but years. Wrinkles and gray hairs are in her case only emblems of maturity, and she never refers to them save to be proud rather than ashamed of them, and no reference to them on the part of others is received as a reflection or an imputation. There are few such, but nearly every one has sometime encountered a character like this.

On the other hand, the number of women who immediately give up all participation in social enjoyments as soon as the first faint phenomena of age approach; who are so eternally conscious of every indication of accumulating years that any reference thereto is construed into an insult or an impertinence; whose every movement and glance seem to be made in dire fear lest some one thinks or is about to speak to others of their symptoms of age; who fret and fume in society, and mope and whine at home because their youth is fading away; who are continually racking their ingenuity in attempts to conceal their age from those who know them to a day; who, in making them selves unhappy, make everybody around them so too; who are talked about and ridiculed by their entire acquaintance, not because they are growing old, but because they are ashamed of it; who are gladly missed from the social circle because of the gloom they bring into it; who are not sympathized with in illness and scarcely mourned when dead; such women are, alas! too numerous in every coterie of society, and the reader can doubtless use up all his or her fingers and thumbs—and toes for that matter—in naming them off.

Isn't it queer that so many women—and even some men—will thus needlessly and voluntarily make more than half of their lives miserable because they are no longer young as they once were. As *Puck* would say: "What fools these mortals be!"

MIXTURE FOR CHAPPED HANDS. (DR. WILSON.)

Carbolic acid, 15 grains.
Yolk of egg, No. 1.
Glycerin, 3 drachms.

A very minute quantity is smeared upon the hands three times a day, provided the skin is not broken.

